# Impact of personality on adherence to and beliefs about ADHD medication, and perceptions of ADHD in adolescents

**DOI:** 10.1186/s12888-020-02543-x

**Published:** 2020-03-30

**Authors:** Maria Emilsson, Per Gustafsson, Gisela Öhnström, Ina Marteinsdottir

**Affiliations:** 1 Department of Health Science, Section of Nursing Graduate Level, University West, 461 86 Trollhättan, Sweden; 2 Center for Social and Affective Neuroscience, Department of Biomedical and Clinical Sciences, Linköping University, Nr 2, 58183 Linköping, Sweden; 3 Department of Child and Adolescent Psychiatry, Linköping, Region Östergötland, Nr 3, 581 85 Linköping, Sweden; 4 Department of Medicine and Optometry, Faculty of Health and Life Sciences, Linnæus University 42157, Kalmar, Sweden

**Keywords:** ADHD, Illness perception, Medication beliefs, Personality traits, Medication adherence

## Abstract

**Background:**

Adherence to attention deficit hyperactivity disorder (ADHD) medication can prevent serious consequences, possibly with lifelong effects. Numerous factors have been observed that influence adherent behaviour, but the impact of personality traits has been inadequately explored. The purpose of this study was to explore the associations between personality traits and adherence to ADHD medication, beliefs about the medication, and perceptions of ADHD.

**Method:**

Adolescents (*n* = 99) on ADHD medication were administered: Health-Relevant Personality Traits Five-Factor Inventory, Medication Adherence Report Scale, Beliefs about Medicines Specific and Brief Illness Perceptions Questionnaires.

**Results:**

The personality trait Antagonism correlated with adherence behaviour (*r* = − 0.198, *p* = 0.005) and perceived personal control of ADHD (*r* = − 0.269, *p* = 0.007). Negative Affectivity correlated with beliefs regarding necessity (*r* = 0.319, *p* = 0.001), concerns (*r* = 0.344, *p* = 0.001), and experienced side effects of medication (*r* = 0.495, *p* = 0.001), alongside perceptions regarding duration (*r* = 0.272, *p =* 0.007), identity (*r* = 0.388, *p* < 0.001), being emotionally affected (*r* = 0.374, *p* < 0.01), personal control (*r* = − 0.287, *p* = 0.004) and concerns about ADHD (*r* = 0.465, *p* < 0.001). Impulsivity correlated with perceived consequences (*r* = − 0.226, *p* = 0.0255) and personal control of ADHD (*r* = − 0.379, *p <* 0.001). Hedonic Capacity correlated with concerns about medication (*r* = − 0.218, *p* = 0.0316) and perceived identification with ADHD (*r* = − 0.203, *p* = 0.045).

**Conclusion:**

Personality traits are related to adherence, beliefs about ADHD medicines and perceptions of ADHD. Antagonism is associated with adherence, especially intentional non-adherence, while Negative Affectivity correlates with numerous perceptions of ADHD and beliefs about medications. Personality assessments could be useful in the care and treatment of adolescents with ADHD.

## Background

Personality traits may influence behaviour through beliefs and attitudes [1], which is an observation that may be important for health services. For instance, adherence is one representation of behaviour in treatment settings [2], which in turn makes personality traits [3] of interest in treatment assessments studies [4, 5].

Personality traits may be defined as “dimensions of individual differences in tendencies to show consistent patterns of thoughts, feelings and actions” (p.25) [1]. One of the most accepted solutions for describing personality traits is the five-factor model (FFM) of personality, also called the “Big Five” [6]. It consists of the following five domains: Neuroticism, Extraversion, Openness to Experience, Agreeableness and Conscientiousness [6].

Interestingly, both low Conscientiousness [7, 8] and high Neuroticism have been associated with poor health-related behaviour [4, 9, 10], such as low adherence [4, 5, 8, 11–13], while high Neuroticism has been associated with more frequent healthcare-seeking [9] and the perception of medicine side effects [14].

With regard to the fact that the five personality traits are supposed to mature up to the age of 30 years [1] and thereafter fewer changes are notable [15], the impact of personality needs to be studied separately in adolescents. More accurately, female Neuroticism increases between 12 and 18 years of age [16], but declines after 21 years of age in both genders [17, 18]. Similarly, Openness to Experience is shown to increase between early and late adolescence [16]. On the other hand, Agreeableness and Conscientiousness are postulated to decrease between 12 and 18 years of age [19, 20] and Extraversion stays the same up to 29 years of age [16, 17, 19].

There is a growing body of knowledge regarding consequences of attention deficit hyperactivity disorder (ADHD) on a person’s life including negative effects on quality of life and sleep [21], risks of ending up in violent interactions [22] and academic preference problems [23]. During adolescence, ADHD symptoms are reported to hinder education, career prospects and social contacts besides increasing the risk of destructive behaviour [24–26]. Hence, it seems especially important to avoid these possibly long-lasting influences of ADHD during adolescence, since it is the period when personal and professional life is founded. Fortunately, medical treatment for individuals with ADHD may reduce some consequences such as substance abuse [27], comorbid psychiatric disorders [28] and criminality [29]. Similarly, adherence to ADHD treatment is reported to be beneficial for achieving academic grades [30], in line with general knowledge about the role of adherence for achieving treatment effects [2].

The reported prevalence rates of adherence to ADHD medication vary widely, with a range of 21–81% in adolescents [30, 31]. In Sweden, we recently reported high adherence, or around 46.5%, in adolescents on longstanding ADHD medication by using the Medication Adherence Report Scale (MARS) [32]. This scale is especially advantageous in clinical settings because it is short and measures both intentional and unintentional non-adherence [33, 34]. Intentional non-adherence is based on an active decision not to take medication as prescribed while unintentional non-adherence is related to capacity and resource limitations in taking the medication; for example, because the medicines are too expensive or due to forgetfulness [33].

Adherence in general is postulated to be a multidimensional phenomenon involving therapy, the healthcare system, diseases and socioeconomic and patient-related factors such as personality traits and the patient’s beliefs [2]. Previously, our group has revealed associations between adherence and beliefs in the necessity for, concerns about and experienced side effects of medications [32] by using the Beliefs about Medicines Questionnaire Specific (BMQ-Specific) in adolescents with ADHD. In addition, non-adherent behaviour that was unintentional [32] was less prominent in individuals who more strongly perceived the consequences of ADHD in everyday life as measured by the Brief Illness Perception Questionnaire (B-IPQ) [35]. B-IPQ was designed for a quick and simple evaluation of illness perceptions [35].

Given that high Neuroticism/negative Emotionality and low Agreeableness and Conscientiousness are associated with non-adherence behaviour in somatic disorders [4, 5, 8, 11–13], and that the very same pattern has been detected and linked to the severity [36] of ADHD in children and adolescents [37], this personality pattern could also be related to adherence to ADHD medication in this group.

In general, there is little research available regarding the influence of personality on illness perception or beliefs about medications [5, 38] and none whatsoever in ADHD. Moreover, to our best knowledge, no literature exists to date regarding the possible impact of personality traits on adherence to medication for the treatment of ADHD.

The Health-Relevant Personality Traits Five-Factor Inventory (HP5i) has some advantages for assessing the role of personality in clinical situations in that it is short and easily filled in and captures the five health-relevant facets of The Big Five [3]. Also, some data are available from Swedish populations because it has previously been employed in Sweden for research on adherence [4] and substance use [39, 40], although not on ADHD.

The aim of this study was to explore possible associations between personality traits and adherence to long-term ADHD medication, beliefs about the medication, and perceptions of ADHD among adolescents. A second aim was to investigate whether personality traits are associated with adherence through beliefs about the medication.

## Methods

### Procedure

Adolescents (aged 13–17 years) who had received ADHD medication for at least 6 months at two Swedish child and adolescent psychiatric clinics (CAP) were offered to take part in the study between March 2014 and June 2015. In addition to language difficulties, exclusions criteria also included autism spectrum-, intellectual developmental- and neurological disorders. Information was enclosed in a letter given prior to a scheduled appointment for a regular follow up which occurred twice a year. After the adolescent and the parent/guardian assigned informed consent, a battery of self-reports was handed out which was comprised of sociodemographic questions, Hp5i [3], MARS [41], BMQ-Specific [42, 43] and B-IPQ [35]. A research nurse (not involved in the clinical treatment) was present while the adolescents filled out the self-reports. An opportunity was given to answer the questionnaires at home if not possible during the visit and in such cases they were returned by mail. In order to ensure independent results and to enable confidentiality, it was explained that the answers would never reach the doctor or any of the staff responsible for treatment. The participation in the study was not rewarded by any kind of compensation.

### Ethical standards

The Regional Ethical Review Board in Linköping (D-nr 2013/402–31) approved this study. The ethical standards of The Helsinki Declaration of 1964 [44] were followed in this research project.

### Participants

Out of the 160 adolescents invited to participate, 148 (92.5%) accepted and 99 (68.9%) completed all questionnaires. Their mean age was 15.6 ***±*** 1.36 years and 64 (64.6%) were males and 35 (35.4%) females. The ADHD diagnosis was based on the DSM-IV criteria and was determined before treatment started by an experienced specialist in child and adolescent psychiatry after a thorough diagnostic investigation. This included neuropsychological and clinical examination, relevant questionnaires and the results of the QbTest (Qbtech. Quantitative behaviour technology. https://www.qbtech.com/. Accessed 26 February 2019), which supported the diagnosis in 93% (92/99) of cases. All adolescents were found to have the combined type of ADHD. The females showed a tendency to be older (*p* = 0.073) and to have been on ADHD medication for a shorter period of time (*p* = 0.068) but the mean time on medication was 51.3 (SD: 29.2) months. The majority, or eighty-one (80.9%) of adolescents had long-acting methylphenidate (MPH) formulations and nine (9.0%) atomoxetine (ATX) added to the ten who had the combination treatment of ATX (10.1%) and MPH. An attrition analysis did not reveal any significant differences regarding gender, age at the start of medication, or time on medication when the 49 non-respondents were compared to the 99 participants [32].

### Questionnaires

#### Health-relevant personality traits five-factor inventory (HP5i)

The HP5i assess five health-relevant facets of the Big Five’s personality traits including; *Negative Affectivity* (a facet of Neuroticism)*, Impulsivity* (an opposite facet to Conscientiousness)*, Hedonic Capacity* (a facet of Extraversion)*, Alexithymia* (an opposite facet to Openness to experiences) and *Antagonism* (an opposite facet to Agreeableness). The inventory has 20 items that are scored between 1 and 4. Each personality trait is covered by four items [3]. The inventory has been validated for Swedish adolescents [39]. In the present study, Cronbach’s alpha was 0.52–0.83.

#### Medication adherence report scale

In order to assess adherent behaviour to prescribed ADHD medication, the MARS questionnaire was used. The first of its five statements evaluates unintentional non-adherence (item 1: “I forgot to take them”) and the remaining four address intentional non-adherence (item 2: “altering the dosage”, item 3: “stop taking medication”, item 4: “missing a dose”, and item 5: “taking less than instructed”) [41]. The items are scored on a 5-point scale capturing the range between “very often” =1 and “never” =5. Low scores on the total MARS questionnaire indicate low adherence behaviour in general while the scores on the 2 subscales define whether it is associated with intentional or unintentional behaviour. Low scores on the “intentional non-adherence” subscale imply more intentional non-adherence behaviour while low scores on the “unintentional non-adherence” subscale are related to behaviour such as forgetting the medication. Cronbach’s alpha for MARS was 0.53 in the present study.

#### Beliefs about medicines questionnaire specific

The BMQ-Specific investigates beliefs about the prescribed medication and consists of eleven questions that are clustered into three subscales. The items are scored on a 5-point scale covering the interval between “strongly disagree” =1 to “strongly agree” =5 [42, 43]. The Specific–Necessity subscale has five questions, and in this study explores beliefs about the necessity of the ADHD medication for relieving symptoms and sustaining health (e.g. “My life would be impossible without my medicines”). The Specific–Concerns subscale has five questions capturing concerns about the negative consequences of the ADHD medication (e.g. “I sometimes worry about the long-term effect of my ADHD medication”). A subtraction of the scale scores of the Specific–Concerns from the Specific–Necessity (range, − 20 to 20) generates the so-called Necessity–Concerns differential. A positive differential score demonstrates that the beliefs in the necessity of the medication are stronger than concerns about its consequences. Conversely, a negative score indicates that the concerns are stronger than the beliefs. The last of the 11 items constitutes the third subscale and assess beliefs about side effects: “I have unpleasant side effects from my ADHD medicines” [42] was not included in the present study. It is not possible to calculate a BMQ-Specific’s total score because the subscales cover dimensions which go into opposite directions, thereby at least partly outweighing each other. Hence, only the scores from the separate subscales and the differential between these subscale scores are used in the statistical analysis. The Cronbach’s alpha was 0.81 for the Specific–Necessity scale and 0.75 for the Specific–Concerns scale.

#### Brief illness perception questionnaire

The adolescents´ perceptions regarding ADHD were investigated using a nine items self-report, B-IPQ. The first eight items of the inventory are scored between 0 and 10 whereas a higher score implies a stronger perception of the respective dimension of ADHD. B-IPQ consists of cognitive dimensions that are measured by five of its items (timeline (chronic vs. acute), identity, consequences, and personal and treatment control of ADHD). In addition, emotional dimensions are assessed by two items (concern about ADHD and being emotionally affected by ADHD) and the last dimension is measured by one item, comprehensibility of ADHD [35]. Finally, item 9 consists of an open question and was not analysed in this study.

#### Statistical analysis

The Statistical Package for the Social Sciences (SPSS) 21.0 was employed for data analysis. Descriptive statistics consisted of frequencies, means and standard deviation calculations. Pearson’s correlation coefficient was utilised to explore associations between personality traits (HP5i) and the variables age, perceptions about ADHD (B-IPQ), beliefs about medication (BMQ-Specific) and adherence behaviour (total MARS questionnaire and un/intentional non-adherence subscales). The effect size for Pearson’s r was classified as small if *r* = 0.1, medium if *r* = 0.3 and large if *r* = 0.5 [45]. The Mann-Whitney’s U-test [45–47] was engaged for analyses of possible gender differences regarding personality traits. The one-sample T-test [45–47] was used to compare personality traits between the adolescents with ADHD aged 16 to 17 years (*n* = 57, males *n* = 34, females *n* = 23) and Swedish normative controls (*n* = 70, males *n* = 29, females *n* = 41) aged 16 to 19 years [48]. A mediation analysis was planned to elucidate whether beliefs about medication mediated the associations between personality traits and adherence, but the demands of the analysis were not fulfilled (see Table 4) [49]. Two stepwise multiple regression models [45–47, 50] were created in which variables were selected according to the following criteria: In the first step, those MARS sub/scales were selected that correlated at least on a *p* level < 0.10 with any of the HP5i personality traits. Two different models were created in which the scores of a) the total MARS questionnaire, and b) intentional subscales were dependent variables. The independent variables in model a) were Antagonism, “the BMQ-Specific-Necessity”, “the BMQ-Specific-Concerns”, “the BMQ-Necessity-Concerns differential” and “the BMQ-unpleasant side-effects”. In model b), the independent variables were as follows: the personality trait of Antagonism, “the BMQ-Specific-Concerns”, “the BMQ-Necessity-Concerns differential” and “the BMQ-unpleasant side-effects”. The effect size for R^2^ was classified as small if R^2^ = 0.02, medium if R^2^ = 0.13 and large if R^2^ = 0.26 [45].

## Results

### In general

Detailed information regarding the results of MARS, BMQ-Specific and B-IPQ are given in a prior publication by Emilsson et al. [32].

### Personality traits

HP5i personality traits were not significantly correlated to age in the ADHD population. The females reported higher Negative Affectivity (*p* < 0.001) and Impulsivity (*p =* 0.019) than the males (Table 1). In comparison with the gender counterparts from a normal population, the females with ADHD reported significantly (*p* = 0.003) higher Impulsivity while the males with ADHD reported significantly (*p* = 0.005) higher Hedonic Capacity.

**Table 1:** Personality traits as measured by HP5i in adolescents with ADHD on long-term medication

	ADHD total	Males with ADHD	Females with ADHD
	(*n* = 99)	(*n* = 64)	(*n* = 35)
	mean (SD)	mean (SD)	mean (SD)
Negative Affectivity^a^	2.26 (0.64)	2.09 (0.58)	2.56 (0.62) ^b^ ***
Antagonism^a^	2.44 (0.83)	2.43 (0.78)	2.46 (0.93) ^b^
Impulsivity^a^	2.65 (0.84)	2.52 (0.78)	2.89 (0.91) ^b^*
Hedonic Capacity^a^	3.13 (0.50)	3.19 (0.45)	3.01 (0.58) ^b^
Alexithymia^a^	2.12 (0.64)	2.16 (0.64)	2.06 (0.64) ^b^

Note. ^a^Health-Relevant Personality Traits Five-Factor Inventory, ^b^Comparison between genders in ADHD population, Mann-Whitney’s U-test, * = *p <* 0.05, ** = *p* ≤ 0.01, *** = *p <* 0.001

### Personality traits and adherence

The HP5i Antagonism scores correlated negatively with scores for the total MARS questionnaire (*p* = 0.050) and its intentional non-adherence subscale (*p =* 0.043). Both findings remained (total MARS questionnaire; *p =* 0.020; intentional non-adherence subscale; *p =* 0.004) in a consecutive gender analysis solely in the males. In addition, Negative Affectivity showed a tendency for a negative correlation with scores for the total MARS questionnaire (*p* = 0.063) and its intentional non-adherence subscale (*p =* 0.058). See Tables 2 and 3.

**Table 2:** Correlations between personality traits (HP5i) and adherence behaviour (MARS), and beliefs about medication (BMQ-Specific) among adolescents (*n* = 99; 64 males and 35 females) with ADHD on long-term medication

	Negative Affectivity^a^			Antagonism^a^			Impulsivity^a^		
	Total	Males	Females	Total	Males	Females	Total	Males	Females
	r	r	r	r	r	r	r	r	r
Total MARS questionnaire^b^	− 0.188	−0.160	− 0.176	− 0.198*	− 0.290*	− 0.104	−0.086	− 0.083	− 0.052
Intentional non-adherence^b^	− 0.191	− 0.162	− 0.163	− 0.204*	− 0.257**	−0.143	− 0.069	− 0.055	−0.034
Unintentional non-adherence^b^	−0.089	− 0.056	− 0.141	− 0.087	− 0.194	0.022	−0.086	− 0.103	−0.073
BMQ-Specific–necessity^c^	0.319***	0.361**	0.088	0.125	0.261*	−0.099	0.048	0.085	− 0.139
BMQ-Specific–concerns^c^	0.344***	0.272*	0.355*	0.153	0.214	0.068	0.087	0.072	0.038
BMQ-Differential score^c^	0.028	0.118	−0.166	− 0.003	0.074	−0.107	− 0.020	0.022	−0.115
BMQ-Side-effects^c^	0.326***	0.339**	0.298	0.092	−0.001	0.211	0.119	0.025	0.213
B-IPQ Consequence^d^	0.182	0.095	0.231	0.004	−0.114	0.214	0.226*	0.129	0.342*
B-IPQ Timeline^d^	0.272**	0.326**	0.145	0.182	0.280*	0.029	0.171	0.125	0.209
B-IPQ Personal Control^d^	−0.287**	− 0.204	− 0.247	− 0.269**	− 0.287*	−0.251	− 0.379**	− 0.224	− 0.535**
B-IPQ Treatment Control^d^	− 0.035	0.009	− 0.228	− 0.158	− 0.196	−0.091	− 0.032	− 0.006	− 0.150
B-IPQ Identity^d^	0.388**	0.484**	0.096	0.178	0.144	0.242	0.146	0.146	0.058
B-IPQ Concern^d^	0.465**	0.311*	0.558**	0.003	− 0.071	0.089	0.144	0.038	0.166
B-IPQ Comprehensibility^d^	− 0.190	− 0.092	− 0.261	− 0.242*	− 0.117	− 0.413*	−0.073	0.027	− 0.157
B-IPQ Emotional response^d^	0.374**	0.378**	0.246	0.331	0.249*	0.102	0.331**	0.300*	0.300

Note. *r* = Pearson’s correlation coefficient ^a^Health-Relevant Personality Traits Five-Factor Inventory, ^b^ Medication Adherence Report Scale, ^c^Beliefs about Medicines Questionnaire-Specific, ^d^The Brief Illness Perception Questionnaire, * = *p <* 0.05, ** = *p ≤* 0.01, *** = *p* ≤ 0.001

**Table 3:** Correlations between personality traits (HP5i) and adherence behaviour (MARS), and beliefs about medication (BMQ-Specific) among adolescents (*n* = 99; 64 males and 35 females) with ADHD on long-term medication

	Hedonic Capacity^a^			Alexithymia^a^		
	Total	Males	Females	Total	Males	Females
	r	r	r	r	r	r
Total MARS questionnaire^b^	0.118	0.113	0.096	−0.091	−0.126	−0.071
Intentional non-adherence^b^	0.145	0.162	0.092	−0.142	−0.182	− 0.116
Unintentional non-adherence^b^	− 0.003	−0.079	0.072	0.078	0.097	0.059
BMQ-Specific–necessity^c^	0.061	0.002	0.252	0.026	0.041	0.047
BMQ-Specific–concerns^c^	−0.218*	−0.238	− 0.147	0.143	0.067	0.296
BMQ-Differential score^c^	0.186	0.156	0.257	−0.072	−0.010	− 0.156
BMQ-Side-effects^c^	− 0.052	−0.022	− 0.059	0.121	0.028	0.280
B-IPQ Consequence^d^	− 0.063	− 0.165	0.161	0.049	0.065	0.054
B-IPQ Timeline^d^	0.084	0.085	0.114	0.160	0.154	0.194
B-IPQ Personal Control^d^	0.068	−0.061	0.157	−0.218*	− 0.263*	−0.211
B-IPQ Treatment Control^d^	0.173	0.228	0.154	−0.036	0.103	−0.292
B-IPQ Identity^d^	−0.203*	−0.172	− 0.216	0.036	0.118	−0.077
B-IPQ Concern^d^	−0.241	− 0.257*	− 0.153	0.019	− 0.050	0.181
B-IPQ Comprehensibility^d^	−0.096	0.044	0.113	−0.030	0.048	−0.180
B-IPQ Emotional response^d^	−0.112	−0.049	− 0.134	−0.132	0.139	0.176

Note. *r* = Pearson’s correlation coefficient, ^a^Health-Relevant Personality Traits Five-Factor Inventory, ^b^ Medication Adherence Report Scale, ^c^Beliefs about Medicines Questionnaire-Specific, ^d^The Brief Illness Perception Questionnaire, * = *p <* 0.05, ** = *p* ≤ 0.01, *** = *p* ≤ 0.001

### Personality traits and beliefs about medication

In the total sample, the HP5i Negative Affectivity scores correlated positively with the BMQ-Specific subscale scores regarding “necessity” (*p =* 0.001), concerns (*p =* 0.001) and “I get unpleasant side-effects from my ADHD medicines” (*p =* 0.001). In a separate gender analysis, Negative Affectivity correlated positively to the necessity scores (*p =* 0.003) and the item “I get unpleasant side-effects from my ADHD medicines” (*p =* 0.006) in males and to the concerns scores in both males (*p =* 0.030) and females (*p =* 0.037). Antagonism correlated positively to the necessity scores (*p* = 0.037) in males. Hedonic Capacity correlated negatively to the concerns scores (*p* = 0.030) in the total group (Tables 2 and 3).

### Personality traits and perceptions of ADHD

The HP5i personality traits were significantly linked to seven of the eight B-IPQ items exploring perceptions of ADHD (see Tables 2 and 3). Negative Affectivity correlated positively to the timeline (*p* = 0.007), identity (*p* < 0.001), concerns (*p* < 0.001) and emotional response (*p* < 0.001), but negatively with the personal control scores (*p* = 0.004). Antagonism was negatively correlated with the personal control (*p* = 0.007) and comprehensibility (*p* = 0.016) scores. Impulsivity correlated negatively with the personal control (*p* < 0.001) and consequences (*p* = 0.025) but positively with emotional response scores (*p* = 0.001). Hedonic Capacity correlated negatively with the identity scores (*p* = 0.045). Alexithymia correlated negatively with personal control (*p* = 0.031) scores.

### Prediction of adherence to ADHD medication

In a stepwise multiple regression model (Table 4), the variance of intentional non-adherence (*R*^2^ = 0.48) was significantly explained by the personality trait Antagonism, along with the necessity-concern differential and BMQ side-effects item. An increment in Antagonism by one point was predictive of a 0.41-point decrease in the intentional non-adherence scores (*p* = 0.047). A one-point increment in the BMQ’s necessity–concern differential was predictive of a 0.11-point increment in the intentional non-adherence (*p* < 0.001). A one-point increase in BMQ’s “experienced side effects” was predictive of a 0.35-point decrease in the intentional non-adherence (*p* = 0.017).

**Table 4:** The variables significantly explaining the variance of a) MARS total questionnaire and b) the intentional non-adherence in two corresponding multiple linear regression models in adolescents (*n* = 99) on long-term ADHD medication

Variables	Total MARS questionnaire ^b^			Intentional non-adherence^b^		
	B	SE B	*P*	B	SE B	*P*
Antagonism^a^	–	–	–	− 0.410	0.203*	0.047
BMQ-differential score^c^	0.140	0.035***	< 0.001	0.107	0.029***	< 0.001
BMQ-Side-effects^c^	−0.430	0.175*	0.016	−0.350	0.144*	0.017

Note. ^a^The Health-Relevant Personality Traits Five-Factor Inventory, ^b^ Medication Adherence Report Scale, ^c^Beliefs about Medicines Questionnaire-Specific, *B* Regression equation, *SE B* Standard Error for B, * = *p <* 0.05, ** = *p ≤* 0.01, *** = *p* ≤ 0.001

In a stepwise multiple regression model, no association was found (*R*^*2*^ = 0.46) between the personality trait Antagonism and the total MARS score (dependent variable) (Table 4). Nevertheless, a one-point increase in the necessity–concern differential increased the total MARS score by 0.14 (*p* < 0.001). A one-point increase in the “experienced side effects” score decreased the total MARS score by 0.43 (*p* = 0.016).

## Discussion

The main results were that the personality trait Antagonism correlated significantly with adherence to ADHD medication by adolescents. Negative Affectivity only showed a tendency to correlate with adherence, despite widespread correlations with perceptions about ADHD and beliefs about medication, including side effects.

These findings provide evidence that personality plays a role in health-related behaviour [1, 51], especially adherence [2]. The association of Antagonism with low adherence is in line with the documented high adherence related to Agreeableness (the opposite of Antagonism) in chronic somatic diseases [11]. More precisely, Antagonism was mainly associated with intentional non-adherence behaviour, defined as the active decision not to take medication as prescribed [33]. This is in agreement with the reported decline in the dimensional opposite of Antagonism and Agreeableness during adolescence [19], suggesting that antagonism may have proportionally more impact during this period. Hence, the previously reported lower adherence among adolescents compared to younger children with ADHD [52, 53] could be partially related to personality influences.

There was no gender difference regarding Antagonism. However, the relation between Antagonism and adherence behaviour was only replicated in males, congruent with findings from young people with asthma [4], suggesting that this relation is not disease-related. Paradoxically, Antagonism was also related to a belief in the necessity of medication only in the male group, raising the question of whether this latter relation is linked to exacerbation of ADHD symptoms due to Antagonism-mediated non-adherence. Further research in this field is needed to elucidate possible links between these two observations.

The results suggest that assessments of personality may be useful in the clinical work to ascertain adherence in young people with ADHD by more directed surveillance and information about the medications for those with high Antagonism.

Other HP5i personality traits showed no significant associations with adherence, which contradicts prior research on somatic disorders, especially regarding Conscientiousness and Neuroticism [4, 5, 8, 11–13]. This discrepancy is probably explained by different age, population and nature of the disorders. However, Negative Affectivity tended to be associated with low adherence to medication in line with findings on people with asthma [5, 11] as well as prior reports showing relation to poor health behaviour in general [9, 10].

Negative Affectivity correlated with numerous beliefs about ADHD medication and perceptions about ADHD, some of which are of interest for adherence [32]. The uneasiness and nervous tension associated with Negative Affectivity probably explain most of the above-described associations [3]. Possibly, these characteristics not only inflate concerns about medication and side effects but also strengthen the belief in the necessity for medication due to worries about ADHD. Furthermore, Negative Affectivity correlated with concerns about ADHD medicines’ side-effects in males. Presumably, this gender difference is related to the medication’s weight-adjustment, which risks proportionally higher concentrations followed by more side effects in males. Interestingly, Negative Affectivity’s associations with experienced side effects, as well as concerns about medication, are in accordance with findings from asthma patients, suggesting that the nature of these associations is to some degree independent of disease. Negative affectivity was also linked to negative perceptions of different aspects of ADHD, comprising long duration, identity with, concerns about, being emotionally affected by and having little personal control over ADHD. Taken together, Negative Affectivity has an impact on the experience of ADHD and medication in a negative direction. This is relevant to consider in clinical work in order to ensure optimal care-taking for promoting individuals’ wellbeing and adherence to medication [32]. Replication studies on larger samples are warranted to further explore the relation between Negative Affectivity and adherence and whether in such cases it is mediated by beliefs about the medication or perceptions about ADHD.

The higher Negative Affectivity (a facet of Neuroticism) and Impulsivity in females with ADHD compared to males parallels research on adolescents with substance misuse problems [40] and has also been described in the general population [54, 55], which suggests a stable gender difference irrespective of populations.

Impulsivity was associated with perceiving more consequences of having ADHD, less personal control over ADHD and being more emotionally affected by ADHD. This is in line with the definition of Impulsivity which describes individuals with limited thoughtfulness, reliability, ability to plan and carefulness [3]. Hence, the personality trait of impulsivity is probably a hinder in the management of ADHD symptoms, which may contribute to more consequences of ADHD.

Higher Hedonic Capacity was accompanied by low concerns about medication, which harmonises with the positive nature of Hedonic Capacity [3].

Several limitations should be mentioned. We cannot exclude the possibility that medication has an impact on the personality outcomes but existing literature is conflicting. Miller et al. [36] detected that personality outcomes were independent of ADHD medication by older teenagers, whereas some influences were noted in an interview study of 13–18-year-old adolescents [56].

Despite giving informed consent, a substantial proportion of the recruited individuals did not return the questionnaires, plausibly due to a tendency for cooperativeness in the presence of caregivers that did not persist after returning home. Furthermore, insufficient support to fill in the questionnaires may have increased the attrition since the caregivers may have similar disabilities. Nevertheless, it is possible that those adolescents who chose not to participate may have more prominent personality deviations. Consequently, the attrition may have limited the study’s ability to elucidate the impact of personality traits on attitudes to ADHD and medication and on adherence [4, 5, 8, 11–13]. On the other hand, the 69% participation rate is acceptable and is probably due to the well-controlled care, which offered good cooperation with both parents/guardians and the research team.

The adherence results may be inaccurate because self-reports such as MARS are indirect assessments that tend to report greater adherence than direct patient observations or pharmacological analyses [33]. In addition, the MARS Cronbach’s alpha only relies on one type of validation, i.e. item inter-correlations [57] and was also of rather low value [58].

This is an exploratory study and therefore no adjustments were made for mass significance, despite the risk of alpha error. In order to avoid overfitting, a stepwise regression was considered more suitable compared to other alternative multiple regression methods, such as the enter [50], forward or backward methods [59].

Since HP5i data from a normal 13–15-year-old population were not available, comparisons with the age- and gender-matched norm population were only performed on participants ≥16 years. Compared to the normal population, the females with ADHD revealed higher Impulsivity while the males with ADHD revealed higher Hedonic Capacity. Higher Impulsivity of the ADHD females is in accordance with previous studies [36, 37]; however, these studies also identified higher Neuroticism and lower Agreeableness but not higher Extraversion (Hedonic Capacity is a facet of Extraversion) [36, 37], compared to our results. This discrepancy is most likely caused by the rather small normal population that we used for comparison, which became more noticeable in the gender comparisons and probably limited the power of the statistical analysis.

A strength of the investigation is the well-characterised sample, because it was possible to identify all suitable patients at the participating clinics, and the solid diagnostic work. The HP5i inventory is validated for Swedish adolescents [39], which excludes cultural influences on the personality results.

## Conclusion

The present results provide evidence for the involvement of personality traits in perceptions about ADHD, beliefs about ADHD medication and adherence in adolescents. Antagonism is associated with adherence to ADHD medication, especially intentional non-adherence behaviour in adolescent males. This suggests that it is important to identify the Antagonism personality trait in the caretaking of ADHD adolescents in order to promote their adherence to medication by means of more information and support.

## Data Availability

Datasets and analyses of the study are not publicly available due to protection of participants’ confidentiality. Anonymised datasets can be created and made available on reasonable requests.

## Abbreviations

ADHD: Attention deficit hyperactivity disorder
ATX: Atomoxetine
B-IPQ: Brief Illness Perception Questionnaire
BMQ-Specific: Beliefs about Medicines Questionnaire Specific
CAP: Child and adolescent psychiatric clinics
FFM: Five-factor model
HP5i: Health-Relevant Personality Traits Five-Factor Inventory
MARS: Medication Adherence Report Scale
MPH: Methylphenidate
QbTest: Qbtech. Quantitative behaviour technology18

## References

[1] McCrae RR, Costa PT (2003). Personality in adulthood : a five-factor theory perspective.

[2] Sabaté E (2003). Adherence to long-term therapies : evidence for action.

[3] Gustavsson JP, Jönsson EG, Linder J, Weinryb RM (2003). The HP5 inventory: definition and assessment of five health-relevant personality traits from a five-factor model perspective. Personal Individ Differ.

[4] Axelsson M, Emilsson M, Brink E, Lundgren J, Toren K, Lötvall J (2009). Personality, adherence, asthma control and health-related quality of life in young adult asthmatics. Respir Med.

[5] Emilsson M, Berndtsson I, Lötvall J, Millqvist E, Lundgren J, Johansson Å, Brink E (2011). The influence of personality traits and beliefs about medicines on adherence to asthma treatment. Prim Care Respir J.

[6] McCrae RR, P T CJ, Boyle GJ, Matthews G, Saklofske DH (2008). Empirical and Theoretical Status of the Five-Factor Model of Personality Traits. The Sage handbook of personality theory and assessment.

[7] Bogg T, Roberts BW (2004). Conscientiousness and health-related behaviors: a meta-analysis of the leading behavioral contributors to mortality. Psychol Bull.

[8] Skinner TC, Bruce DG, Davis TME, Davis WA (2014). Personality traits, self-care behaviours and glycaemic control in type 2 diabetes: the Fremantle diabetes study phase II. Diabet Med.

[9] Costa PT, McCrae RR (1987). Neuroticism, somatic complaints, and disease: is the bark worse than the bite?. J Pers.

[10] Otonari J, Nagano J, Morita M, Budhathoki S, Tashiro N, Toyomura K, Kono S, Imai K, Ohnaka K, Takayanagi R (2012). Neuroticism and extraversion personality traits, health behaviours, and subjective well-being: the Fukuoka study (Japan). Qual Life Res.

[11] Axelsson M, Brink E, Lundgren J, Lotvall J (2011). The influence of personality traits on reported adherence to medication in individuals with chronic disease: an epidemiological study in West Sweden. PLoS One.

[12] Cheung MM, LeMay K, Saini B, Smith L (2014). Does personality influence how people with asthma manage their condition?. J Asthma.

[13] Axelsson M (2013). Report on personality and adherence to antibiotic therapy: a population-based study. BMC Psychol.

[14] Foster JM, Sanderman R, van der Molen T, Mueller T, van Sonderen E (2008). Personality influences the reporting of side effects of inhaled corticosteroids in asthma patients. J Asthma.

[15] McCrae RR, Costa PT, Ostendorf F, Angleitner A, Hrebickova M, Avia MD, Sanz J, Sanchez-Bernardos ML, Kusdil ME, Woodfield R (2000). Nature over nurture: temperament, personality, and life span development. J Pers Soc Psychol.

[16] McCrae RR, Costa PT, Terracciano A, Parker WD, Mills CJ, De Fruyt F, Mervielde I (2002). Personality trait development from age 12 to age 18: longitudinal, cross-sectional, and cross-cultural analyses. J Pers Soc Psychol.

[17] Wängqvist M, Lamb ME, Frisén A, Hwang CP (2015). Child and adolescent predictors of personality in early adulthood. Child Dev.

[18] Terracciano A, McCrae RR, Brant LJ, Costa PT (2005). Hierarchical linear modeling analyses of the NEO-PI-R scales in the Baltimore longitudinal study of aging. Psychol Aging.

[19] Allik J, Laidra K, Realo A, Pullmann H (2004). Personality development from 12 to 18 years of age: changes in mean levels and structure of traits. Eur J Personal.

[20] De Fruyt F, Bartels M, Van Leeuwen KG, De Clercq B, Decuyper M, Mervielde I (2006). Five types of personality continuity in childhood and adolescence. J Pers Soc Psychol.

[21] Peasgood T, Bhardwaj A, Biggs K, Brazier JE, Coghill D, Cooper CL, Daley D, De Silva C, Harpin V, Hodgkins P (2016). The impact of ADHD on the health and well-being of ADHD children and their siblings. Eur Child Adolesc Psychiatry.

[22] Stefanini JR, Scherer ZAP, Scherer EA, Cavalin LA, Guazzelli MS (2015). Adolescents with attention deficit hyperactivity disorder and exposure to violence: parents’ opinion. Rev Lat Am Enfermagem.

[23] Birchwood J, Daley D (2012). Brief report: the impact of attention deficit hyperactivity disorder (ADHD) symptoms on academic performance in an adolescent community sample. J Adolesc.

[24] Molina BS, Pelham WE, Gnagy EM, Thompson AL, Marshal MP (2007). Attention-deficit/hyperactivity disorder risk for heavy drinking and alcohol use disorder is age specific. Alcohol Clin Exp Res.

[25] Turgay A, Goodman DW, Asherson P, Lasser RA, Babcock TF, Pucci ML, Barkley R (2012). Lifespan persistence of ADHD: the life transition model and its application. J Clin Psychiatry.

[26] Barkley RA, Fischer M, Smallish L, Fletcher K (2006). Young adult outcome of hyperactive children: adaptive functioning in major life activities. J Am Acad Child Adolesc Psychiatry.

[27] Chang Z, Lichtenstein P, Halldner L, D'Onofrio B, Serlachius E, Fazel S, Langstrom N, Larsson H (2014). Stimulant ADHD medication and risk for substance abuse. J Child Psychol Psychiatry.

[28] Biederman J, Monuteaux MC, Spencer T, Wilens TE, Faraone SV (2009). Do stimulants protect against psychiatric disorders in youth with ADHD? A 10-year follow-up study. Pediatrics.

[29] Lichtenstein P, Larsson H (2013). Medication for attention deficit-hyperactivity disorder and criminality. N Engl J Med.

[30] Marcus SC, Durkin M (2011). Stimulant adherence and academic performance in urban youth with attention-deficit/hyperactivity disorder. J Am Acad Child Adolesc Psychiatry.

[31] Ferrin M, Taylor E (2011). Child and caregiver issues in the treatment of attention deficit-hyperactivity disorder: education, adherence and treatment choice. Future Neurol.

[32] Emilsson M, Gustafsson PA, Ohnstrom G, Marteinsdottir I. Beliefs regarding medication and side effects influence treatment adherence in adolescents with attention deficit hyperactivity disorder. Eur Child Adolesc Psychiatry. 2016;25(5):559–71. 10.1007/s00787-016-0919-1 PMC5394130 27848023

[33] Horne R, Clatworthy J: Adherence to Advance and Treatment. In: Health Psychology*.* 2nd edn. Edited by French D, Society BP. Chichester, West Sussex Leicester: Wiley-Blackwell. Br Psychol Soc. 2010. p. 175–88.

[34] Lehmann A, Aslani P, Ahmed R, Celio J, Gauchet A, Bedouch P, Bugnon O, Allenet B, Schneider MP (2014). Assessing medication adherence: options to consider. Int J Clin Pharm.

[35] Broadbent E, Petrie KJ, Main J, Weinman J (2006). The brief illness perception questionnaire. J Psychosom Res.

[36] Miller CJ, Miller SR, Newcorn JH, Halperin JM (2008). Personality characteristics associated with persistent ADHD in late adolescence. J Abnorm Child Psychol.

[37] Martel MM, Nigg JT, Lucas RE (2008). Trait mechanisms in youth with and without attention-deficit/hyperactivity disorder. J Res Pers.

[38] Axelsson M, Cliffordson C, Lundback B, Lötvall J (2013). The function of medication beliefs as mediators between personality traits and adherence behavior in people with asthma. Patient Prefer Adherence.

[39] Hemphälä M, Gustavsson JP, Tengstrom A (2013). The validity of the health-relevant personality inventory (HP5i) and the junior temperament and character inventory (JTCI) among adolescents referred for a substance misuse problem. J Pers Assess.

[40] Gunnarsson M, Petter Gustavsson J, Tengström A, Franck J, Fahlke C (2008). Personality traits and their associations with substance use among adolescents. Personal Individ Differ.

[41] Horne R, Hankins M (2004). The medication adherence report scale (MARS).

[42] Horne R, Weinman J (1999). Patients' beliefs about prescribed medicines and their role in adherence to treatment in chronic physical illness. J Psychosom Res.

[43] Horne R, Weinman J, Hankins M (1999). The beliefs about medicines questionnaire: the development and evaluation of a new method for assessing the cognitive representation of medication. Psychol Health.

[44] World Medical Association of Helsinki (2008). World Medical Association declaration of Helsinki : ethical principles for medical research involving human subjects.

[45] Field A (2012). Discovering statistics using IBM SPSS statistics, 4 edn: Saga.

[46] Altman DG (1991). Practical statistics for medical research.

[47] Pallant J (2007). SPSS survival manual: a step-by-step guide to data analysis using SPSS version 15.

[48] Gunnarsson M, Gustavsson P. Normativ data för HP5i baserat på SOM undersökningen 2011. Göteborg; 2013.

[49] Hayes AF (2013). Introduction to mediation, moderation, and conditional process analysis : a regression-based approach.

[50] Tabachnick BG, Fidell LS (2013). Using multivariate statistics.

[51] Booth-Kewley S, Vickers RR (1994). Associations between major domains of personality and health behavior. J Pers.

[52] Sanchez RJ, Crismon ML, Barner JC, Bettinger T, Wilson JP (2005). Assessment of adherence measures with different stimulants among children and adolescents. Pharmacother.

[53] Gau SS, Shen HY, Chou MC, Tang CS, Chiu YN, Gau CS (2006). Determinants of adherence to methylphenidate and the impact of poor adherence on maternal and family measures. J Child Adolesc Psychopharmacol.

[54] Costa P, Terracciano A, McCrae RR (2001). Gender differences in personality traits across cultures: robust and surprising findings. J Pers Soc Psychol.

[55] Lynn R, Martin T (1997). Gender differences in extraversion, neuroticism, and psychoticism in 37 nations. J Soc Psychol.

[56] Brinkman WB, Sherman SN, Zmitrovich AR, Visscher MO, Crosby LE, Phelan KJ, Donovan EF (2012). In their own words: adolescent views on ADHD and their evolving role managing medication. Acad Pediatr.

[57] Connelly LM (2011). Research roundtable. Cronbach’s Alpha. Medsurg Nurs.

[58] Bland JM, Altman DG (1997). Cronbach’s alpha. BMJ (Clinical research ed).

[59] Zar JH (2010). Biostatistical analysis.

